# Time-resolved electron holography and its application to an ionic liquid specimen

**DOI:** 10.1093/jmicro/dfad003

**Published:** 2023-01-11

**Authors:** Yoh Iwasaki, Zentaro Akase, Keiko Shimada, Ken Harada, Daisuke Shindo

**Affiliations:** Center for Emergent Matter Science, Institute of Physical and Chemical Research, 2-1 Hirosawa, Wako, Saitama 351-0198, Japan; Institute of Multidisciplinary Research for Advanced Materials, Tohoku University, 2-1-1 Katahira, Aoba-ku, Sendai, Miyagi 980-8577, Japan; Center for Emergent Matter Science, Institute of Physical and Chemical Research, 2-1 Hirosawa, Wako, Saitama 351-0198, Japan; Center for Emergent Matter Science, Institute of Physical and Chemical Research, 2-1 Hirosawa, Wako, Saitama 351-0198, Japan; Center for Emergent Matter Science, Institute of Physical and Chemical Research, 2-1 Hirosawa, Wako, Saitama 351-0198, Japan

**Keywords:** electron holography, transmission electron microscopy, time resolution, stroboscopic, pump probe, ionic conductor

## Abstract

Time-resolved electron holography was implemented in a transmission electron microscope by means of electron beam gating with a parallel-plate electrostatic deflector. Stroboscopic observations were performed by accumulating gated electron interference images while applying a periodic modulation voltage to a specimen. Electric polarization in an ionic liquid specimen was observed under applied fields. While a static electric field in the specimen was reduced by the polarization of the material, an applied field modulated at 10 kHz was not screened. This indicates that time-resolved electron holography is capable of determining the frequency limit of dynamic response of polarization in materials.

**Graphical Abstract** 
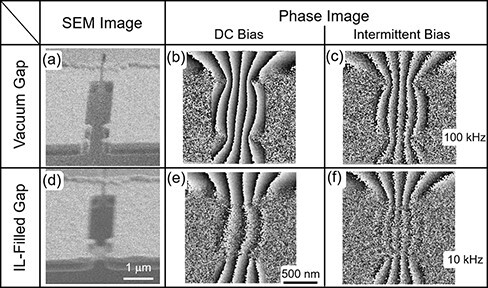

Time-resolved (T-R) observation has been a major trend in transmission electron microscopy (TEM) [1,2]. Time resolution has been drastically improved by electron pulses produced by laser-driven cathodes [3]. Using pump-probe technique with the electron pulses, a variety of dynamic behaviors of materials have been revealed with different TEM imaging methods: phonon vibrations in crystals have been studied using bright-field imaging [4] and the motions of magnetic skyrmions have been studied using the Lorentz method [5,6].

Electron holography (EH), one of the TEM methods, possesses unique sensitivity to electromagnetic potentials and has been applied to a variety of specimens [7,8]. T-R versions of EH have also been intensively studied [9–11], whereas application to materials characterization has been rare. A pioneering T-R EH application to materials was reported on LiCoO_2_ and a Li^+^-conducting solid electrolyte under cyclic voltage modulation [12]. In this experiment, a magnetic scanning coil was used to gate the electron beam (e-beam) and enabled an exposure duty ratio of 47% at 37.6 kHz repetition. The higher duty ratio of exposure helped shorten the overall acquisition time in the T-R EH experiment.

In EH imaging, e-beam convergence on the specimen is avoided in order to achieve higher electron wave coherence. Unfortunately, thus reduced illuminance leads to a longer acquisition time, which causes drift problems even in EH with continuous exposure [13]. The need for an augmented acquisition time, especially in T-R observations with lowered exposure duty ratio, has made T-R EH trials more difficult. Beside the laser-driven cathodes, techniques to deflect e-beam by radio frequency electric waves have been reported as gating methods for T-R TEM [14–16]. While these devices enabled higher exposure duty ratio, compatibility with EH has not become clear yet. The purpose of our present study was 2-fold: to implement T-R EH with a convenient value of exposure duty ratio and to demonstrate its capability in characterizing the dynamic properties of materials.

Direct detection cameras for TEM can currently acquire >1000 frames per second. While a K3 direct electron detector (Gatan) operates internally at 1500 Hz [17], the corresponding exposure time per frame is 1/1500 s = 667 μs, which is insufficient for achieving a time resolution of a few μs or less. As an additional ‘shutter’ function to gate the e-beam, we deflected the beam out of the field of view by applying an electrostatic field. The electrostatic deflector was expected to work faster than magnetic coils installed in the TEM for e-beam steering or scanning.

As shown in Fig. 1, the experimental setup consisted of a TEM and a signal generator with two output channels. The TEM (HF3300X, Hitachi High Technologies Co.) was equipped with a cold cathode field emitter and was operated at an acceleration voltage of 300 kV. An electron source generated a continuous e-beam, an electrostatic beam deflector located immediately after the condenser lens aperture functioned as a fast shutter and a double-biprism configuration located downstream of the specimen produced optical interference [18]. Displacement of the beam spot on the object plane per applied voltage on the parallel-plate deflector was 2.1 μm/V, and only a few volts was sufficient to move the beam completely out of the field of view. Note that the in-focus image of the object on the camera image plane does not shift when the beam is tilted, whereas the image of interference fringes does shift [13].

**Fig. 1.**
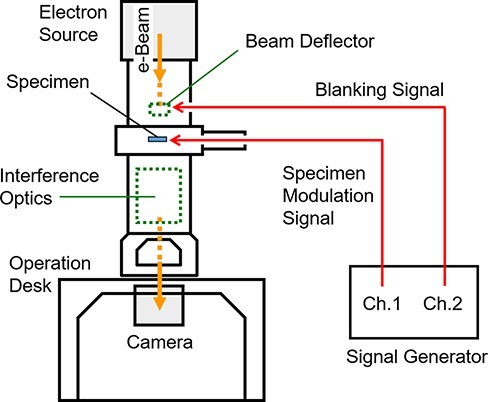
Experimental setup consisting of transmission electron microscope with electrostatic beam deflector and interference optics and signal generator.

A modulation voltage from a signal generator (WF1968, NF Corporation) was applied to the specimen stage, and a blanking voltage was applied to the deflector from the other output channel of the signal generator. The outputs of the two channels were synchronized and shifted by a certain phase in the time repetition period. Exposures within a limited window in every modulation period were repeated for a large number of cycles to accumulate a sufficient number of electrons on the CCD camera (US4000, Gatan). Stroboscopic T-R EH imaging, or pump-probe observation, was enabled in this manner. Although this type of accumulation can image only repetitively reversible phenomena and overlooks responses that change sporadically cycle by cycle, ‘this methodology is highly effective for investigating the intrinsic mean motions’ of the target material [6].

First, interference fringes of electron waves traveling in vacuum (without a specimen present) were imaged by stroboscopic exposure, and the visibility of the fringes was measured as a function of the width of a single exposure. As shown in Supplementary Material 1, the visibility of the fringes imaged with an exposure window >5 μs was comparable to that obtained by conventional continuous exposure. Since the interference fringes on the image plane shifted with beam tilting, the decreased visibility with a shorter exposure window is mainly attributed to superposition of the traversing fringes on the camera during the course of deflector transition (see Supplementary Material 2).

To obtain T-R EH images of an electric field changing over time, a specimen consisting of two Au electrodes was fabricated by means of focused ion beam (FIB) etching. The configuration of the specimen is shown in Supplementary Material 3 with an illustration of electrical connection. To suppress charging of the insulating Si-N substrate supporting the electrodes by electron irradiation, a thin W layer was deposited around the electrodes. Rectangular pulses (4 V in amplitude, 10 μs in length and with a repetition period of 100 μs) were cyclically applied via a Schottky barrier diode to charge one of the electrodes. During each cycle after the 4 V charging was switched off, the electric field decreased over time as clearly shown in the series of snapshots with 5 μs exposure window, i.e. 5% duty ratio of repetition period, in Fig. 2.

**Fig. 2.**
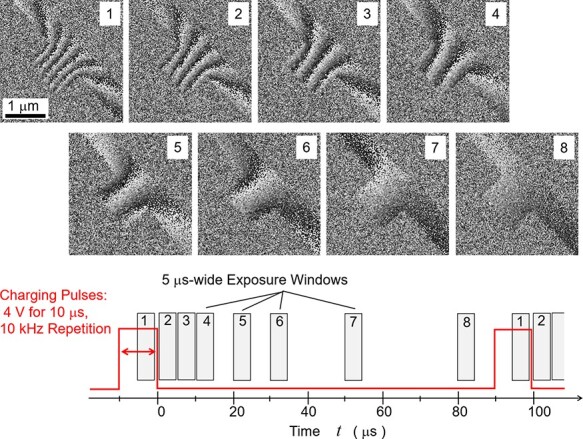
Stroboscopic phase images of discharging electrodes. A dark to bright band corresponds to a 2π phase.

The covering of the Si-N surface with the thin W layer resulted in an unintended contribution to the electric field due to the residual charging of the resistive parts on the specimen. To observe the effect of an intentionally applied voltage more clearly, we calculated the difference between phase distributions obtained with and without intentional biasing (Supplementary Material 4). Each phase image in Fig. 2 was obtained after a reference phase distribution obtained under zero bias with continuous exposure was subtracted.

To evaluate the capability of T-R EH in characterizing the dynamic response of materials, we used an ionic conductor as the target material. Ionic liquids (ILs), a class of ionic conductors, possess low vapor pressure compatible with a specimen chamber for electron microscopy, and EH observations of ILs have been reported with image acquisition by conventional continuous exposures [19,20]. An IL, N,N,N-trimethyl-N-propylammonium bis (trifluoromethanesulfonyl) imide [21] was used. A gap between Au electrodes was prepared by FIB etching, and two Au protrusions were fabricated on each electrode so that the liquid film of the IL was supported across the gap in the middle of the four protrusions, as shown in the scanning electron microscopy (SEM) images in Fig. 3. To reconstruct a phase image under the applied voltage, a hologram obtained under +2 V conditions was processed using a hologram obtained under −2 V conditions as a reference, which enabled a phase gradient corresponding to 4 V to be obtained while suppressing electrolysis of the IL with reduced absolute voltage of 2 V across the IL. Phase images, or potential distributions, were compared focusing on two aspects: IL-filled gap vs. vacuum gap and under periodically changing voltage vs. under constant DC bias.

**Fig. 3.**
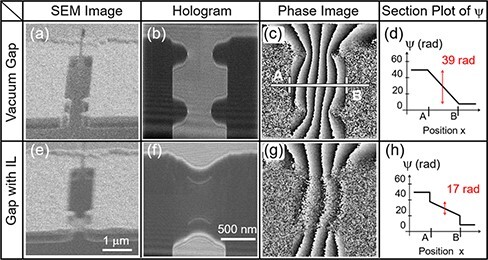
SEM images (a, e), holograms (b, f) and phase images (c, g) of the specimen before and after IL filling of gap. SEM images were observed at 58° off from the substrate normal. Holograms and phase images were obtained under constant bias voltage. Phase distributions across the gap (along the line, A–B, indicated in (c)) are schematically illustrated in (d, h).

IL, ionic liquid; SEM, scanning electron microscopy.

A comparison of the phase distributions in Fig. 3c and Fig. 3 reveals that the number of bands (dark to bright corresponds to a 2π phase) in the gap was remarkably reduced (almost halved) when the gap was filled with IL while the numbers of bands remained the same outside the gap. Recalling the fact that externally applied field is screened inside a conducting material, the reduction of the phase gradient across the gap in Fig. 3g can be readily understood. Note that some of the bands outside the gap in Fig. 3g are not continuous through the gap unlike the continuous bands in Fig. 3c. This indicates that steep phase gradients existed in the electric bilayers formed at the interface between the electrolyte and Au electrodes and that they were thinner than the spatial resolution of the phase image. The schematic section plots of the phase, Ψ, illustrated in Fig. 3d and Fig. 3 are drawn based on the measured phase differences across the gap and on the assumption that phase jumps at the electric bilayers are the same on both sides of the gap. As shown in the SEM image in Fig. 3e, the IL did not fill the entire space between the electrodes, which is consistent with an incomplete canceling of the field in the gap with IL.

Stroboscopic images were acquired when rectangular wave between +2 V and −2 V was applied to the specimen gap. As illustrated in Fig. 4c, the duty ratio of the wave was 50%; stroboscopic holograms were accumulated synchronously within the +2 V application periods. Phase images were reconstructed using reference holograms of respective specimens acquired by continuous exposure under a constant bias of −2 V.

**Fig. 4.**
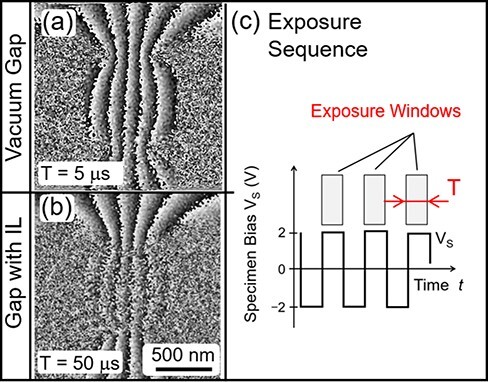
Phase images (a, b) of the specimen under alternating bias voltages before and after IL filling of gap. Rectangular voltage waveform and exposure windows are shown in (c).

IL, ionic liquid.

The stroboscopic phase image of the vacuum gap in Fig. 4a shows the same number of bands as that in Fig. 3c, which was obtained under a constant bias. This indicates that the voltage across the gap followed the rectangular wave output of the signal generator without significant distortion during the stroboscopic exposure window (5 μs each with 100 kHz repetition).

The last experiment was T-R EH observation of the IL under modulation voltages. Figure 4b shows a stroboscopic phase image of the IL-filled specimen obtained in the same manner but with lower repetition frequency of 10 kHz and with an individual exposure window of 50 μs. This phase image was reconstructed from a hologram accumulated in only 6 s, which was enabled by the high exposure duty ratio of 50% in the repetition period. This stroboscopic phase image is distinctly different from that of the same IL-filled specimen obtained under a constant bias (Fig. 3g), and it more closely resembles the phase distributions observed with the vacuum gap where the field consistently followed the externally applied voltages. The same number of bands as in the IL-filled gap in Fig. 4b can be seen in the phase images of the vacuum gap (Figs. 3c and 4a). This indicates that the polarization in the IL did not cancel the externally applied field in this last experiment. In other words, the polarization in the IL did not respond to changes in the external field at this rate. A modulation frequency of 10 kHz that yielded the unresponsive phase distribution is considered as an upper bound for the supremum frequency of the dynamic response of the dominant component of electric polarization. At this low frequency range, the dominant contribution is macroscopic polarization in the specimen built up by the motion of ions across the gap [22]. A series of extended T-R EH observations with varied modulation frequency would locate the supremum frequency and provide information that can be compared with electric impedance spectroscopy of the IL material.

In summary, we implemented T-R EH with a periodic gating by electrostatically deflecting the continuous e-beam. A gated exposure window of ≥5 μs was usable with a duty ratio of 2%–50% (tunable) in the repetition period. Stroboscopic, or pump-probe, T-R EH observations of electric polarization were performed with a modulation voltage applied to the specimen. The present method of gating turned out to be advantageous in that a T-R hologram can be obtained within a practically short acquisition time owing to the far higher exposure duty ratio, which cannot be obtained by using laser-driven electron pulses. By analyzing stroboscopic phase images, information on the upper frequency limit of an electrical response was obtained with an IL specimen. T-R EH is capable of characterizing dynamic electrical responses in ionic conductors.

## Supplementary Material

dfad003_Supp
