# Supplementary material for: Time-resolved electron holography and its application to an ionic liquid specimen
Source: Microscopy (Oxf). 2023 Jan 11;72(5):455–9. doi: 10.1093/jmicro/dfad003 (PMC10561666; doi:10.1093/jmicro/dfad003)
Supplement: dfad003_Supp [file dfad003_supp.zip › suppl_data/230106_Supplementary_Materials.docx]

Supplementary Material 1


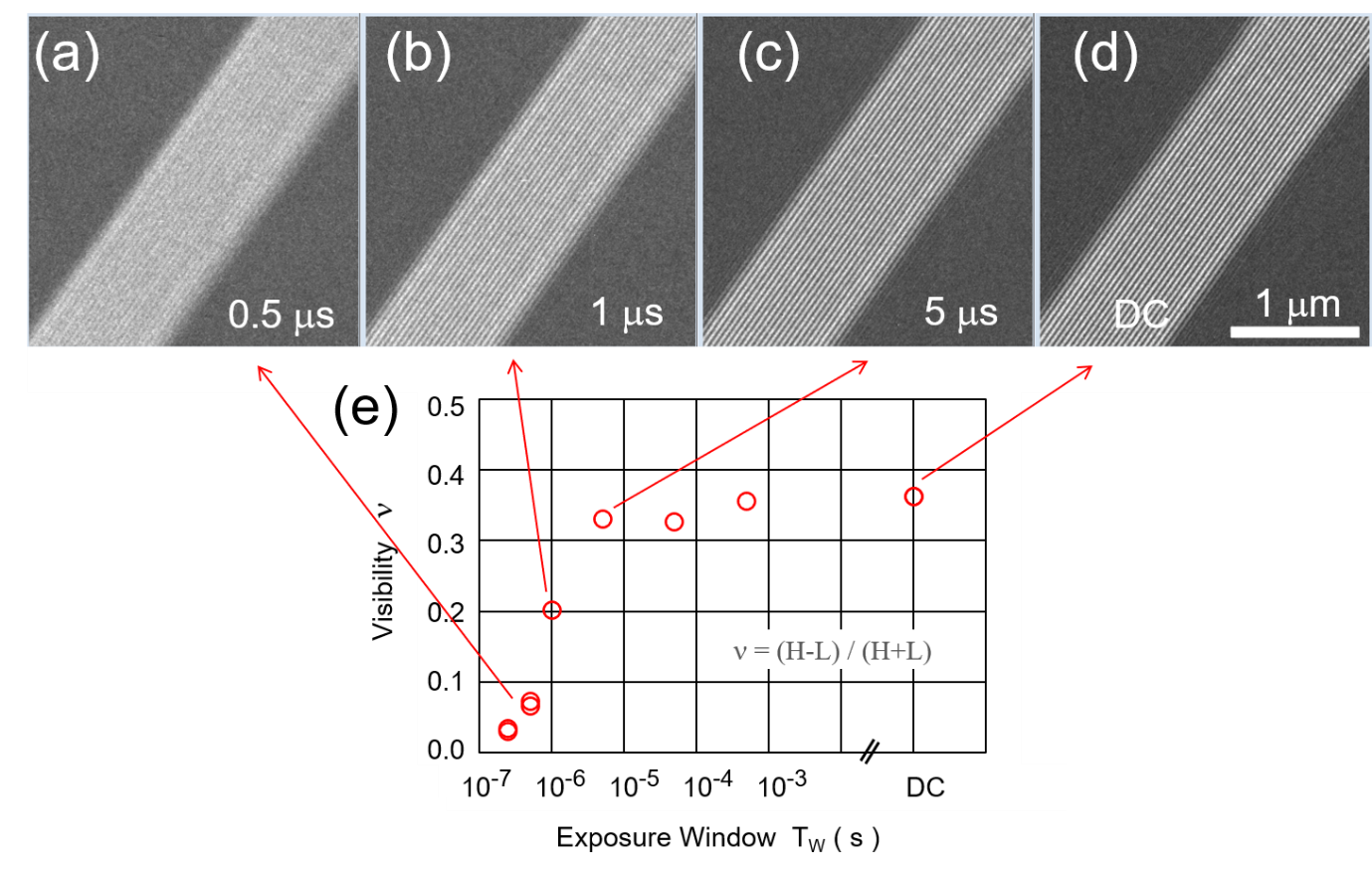


Fig. S1. Visibility of interference fringes vs. exposure window.

Interference fringes were imaged by repeated stroboscopic exposures, (a) to (c), and by continuous exposure, (d). The duty ratio of exposure within the repetition period was commonly 50 %. As the single exposure time became shorter, the total number of repetitions was increased so that the accumulated net exposure time was 4 s for all images. The visibilities of the fringes are plotted in (e) as a function of the individual exposure window width.

Since the interference fringes shift with beam tilting, images of traversing fringes are superposed during a short but finite length of time in the course of beam deflection. The decreased visibility with exposure windows of 1 μs or less is attributed mainly to the increased contribution of the traversing fringes in the narrower exposure windows.

Supplementary Material 2


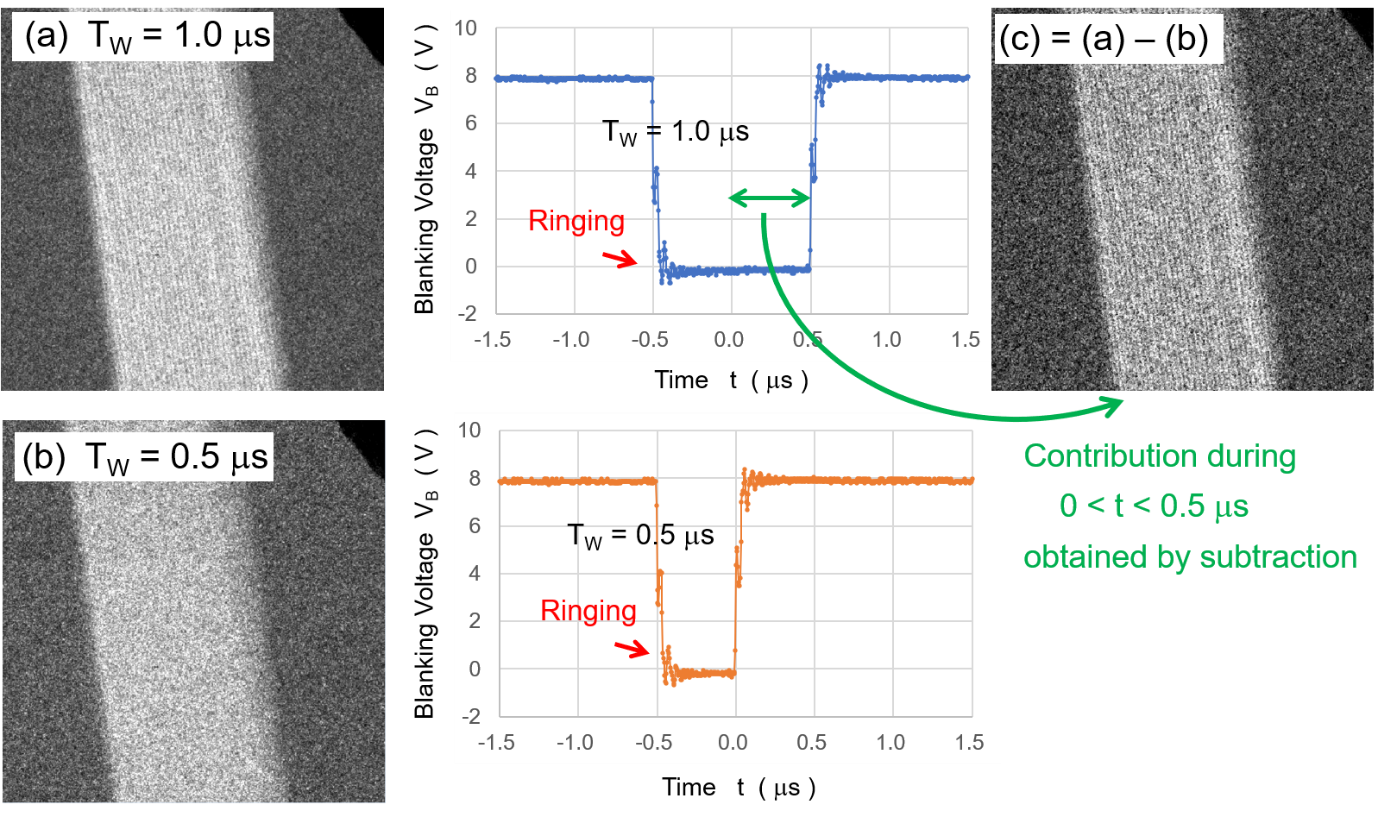


Fig. S2. Interference fringes and deflector voltage waveforms.

Beam deflection voltage waveforms with present deflector device are shown for exposure windows of 1 μs and 0.5 μs. After the voltage transition started at *t* = -0.5 μs, remarkable ringing continued for about 0.1 μs before the voltage settled to zero. The beam tilting during the ringing period resulted in accumulation of shifted interference fringe images in the CCD camera. Since the proportion of this transition period was less than 0.02 for exposure windows of 5 μs or longer, contribution of shifted image was not significant in the integrated image as observed in Supplementary Material 1. Contrarily, as the exposure window became narrower, image of interference fringes degraded as in the images (a) and (b) above.

Subtracting image (b) from image (a) yielded image (c) as a contribution from the period, 0 < *t* < 0.5 (μs), with much clearer fringes than in image (a) or (b). This is an impressive demonstration of the effect of the transition period on the hologram, which may help understand the visibility vs. exposure window relation presented in Supplementary Material 1.

Supplementary Material 3


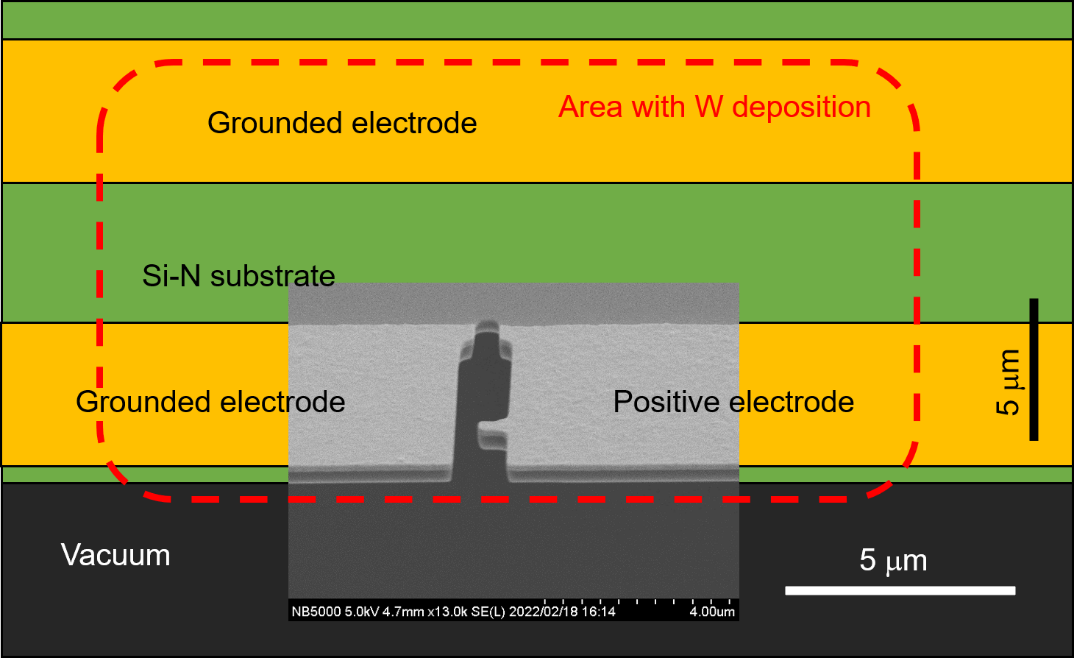


Fig. S3-1. Geometry of the specimen observed at 58° off from the substrate normal.

A scanning electron microscopy (SEM) image is shown in Fig. S3-1. A 1.2-μm-wide vacuum gap was fabricated using a focused Ga ion beam in the middle of a 5-μm-wide Au film formed along the edge of a Si-N substrate. The Au films separated by the gap served as a grounded electrode and a biased electrode. To reduce charging of the exposed Si-N substrate by electron irradiation, electrical conduction was provided on the Si-N surface by depositing a thin layer of W to cover the TEM field of view.


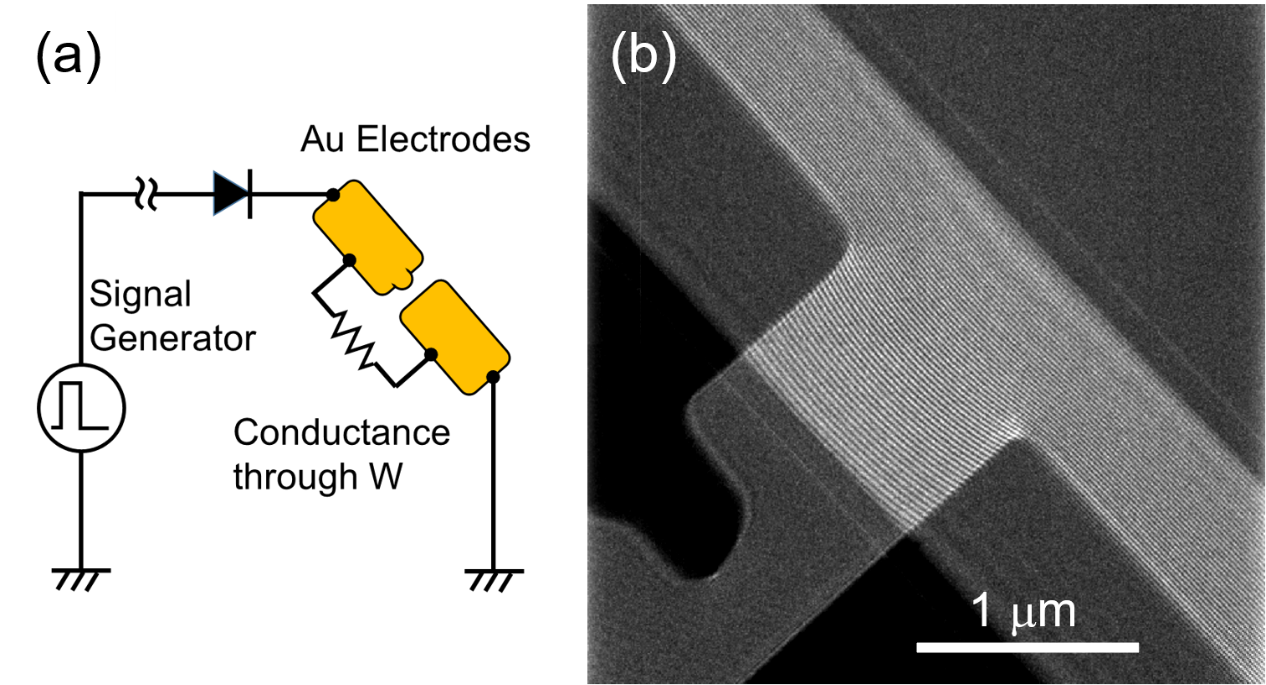


Fig. S3-2. Electrical connection, (a), and a hologram, (b).

A Schottky barrier diode was located next to the specimen as shown in Fig. S3-2(a), and one of the electrodes was charged positively via the diode. As the output voltage of the signal generator decreased, the switching function of the diode separated the charged specimen from the external circuit, allowing the voltage between the electrodes to autonomously decrease with discharge through the thin W layer on the Si-N substrate.

Holograms (images of interference fringes) were obtained so that the interference fringes covered the edge region of the gap between Au electrodes, as shown in Fig. S3-2(b).

Supplementary Material 4


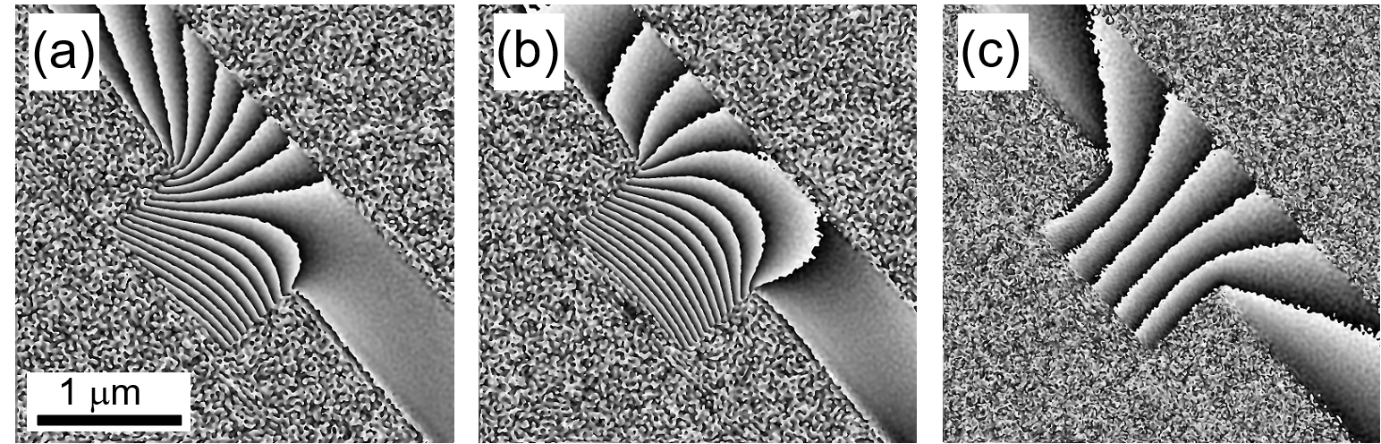


Fig. S4. Phase images around the vacuum gap under a DC bias voltage.

Phase images, (a) and (b), were reconstructed from holograms obtained with applied DC bias voltages of 4 V and 0 V, respectively. Even in the phase image with zero biasing, (b), a phase gradient along the depth of the gap is evident, which indicates charging of the interior part (naked Si-N substrate area is exposed far from the edge) of the specimen. Phase image, (c), represents difference between phase distributions (a) and (b). It shows a uniform phase gradient as expected between the two Au electrodes.
