# Supplementary figures and images for: Time-resolved electron holography and its application to an ionic liquid specimen
Source: Microscopy (Oxf). 2023 Jan 11;72(5):455–9. doi: 10.1093/jmicro/dfad003 (PMC10561666; doi:10.1093/jmicro/dfad003)

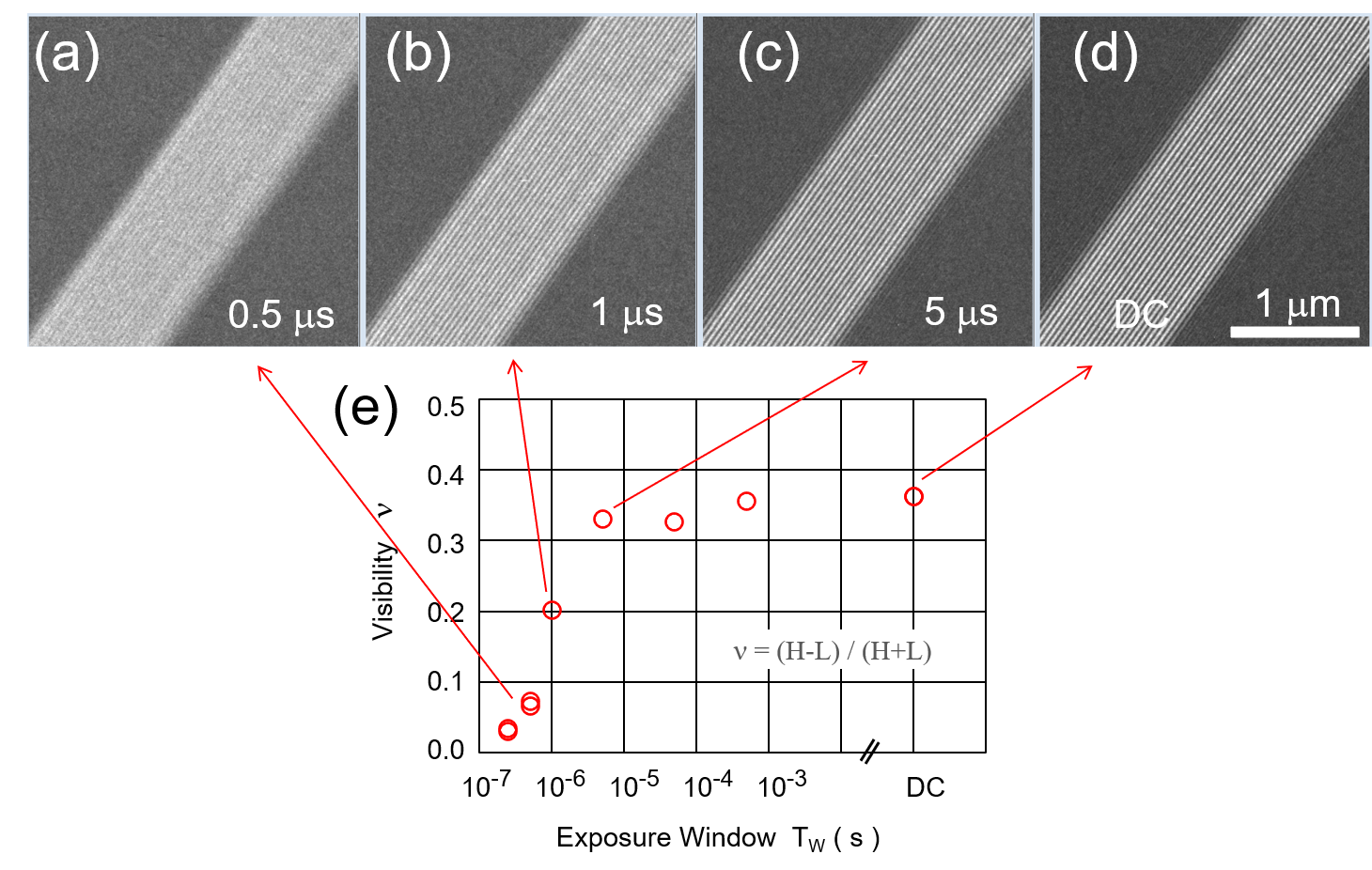

Supplement: dfad003_Supp [file dfad003_supp.zip › suppl_data/Fig. S1.tif]

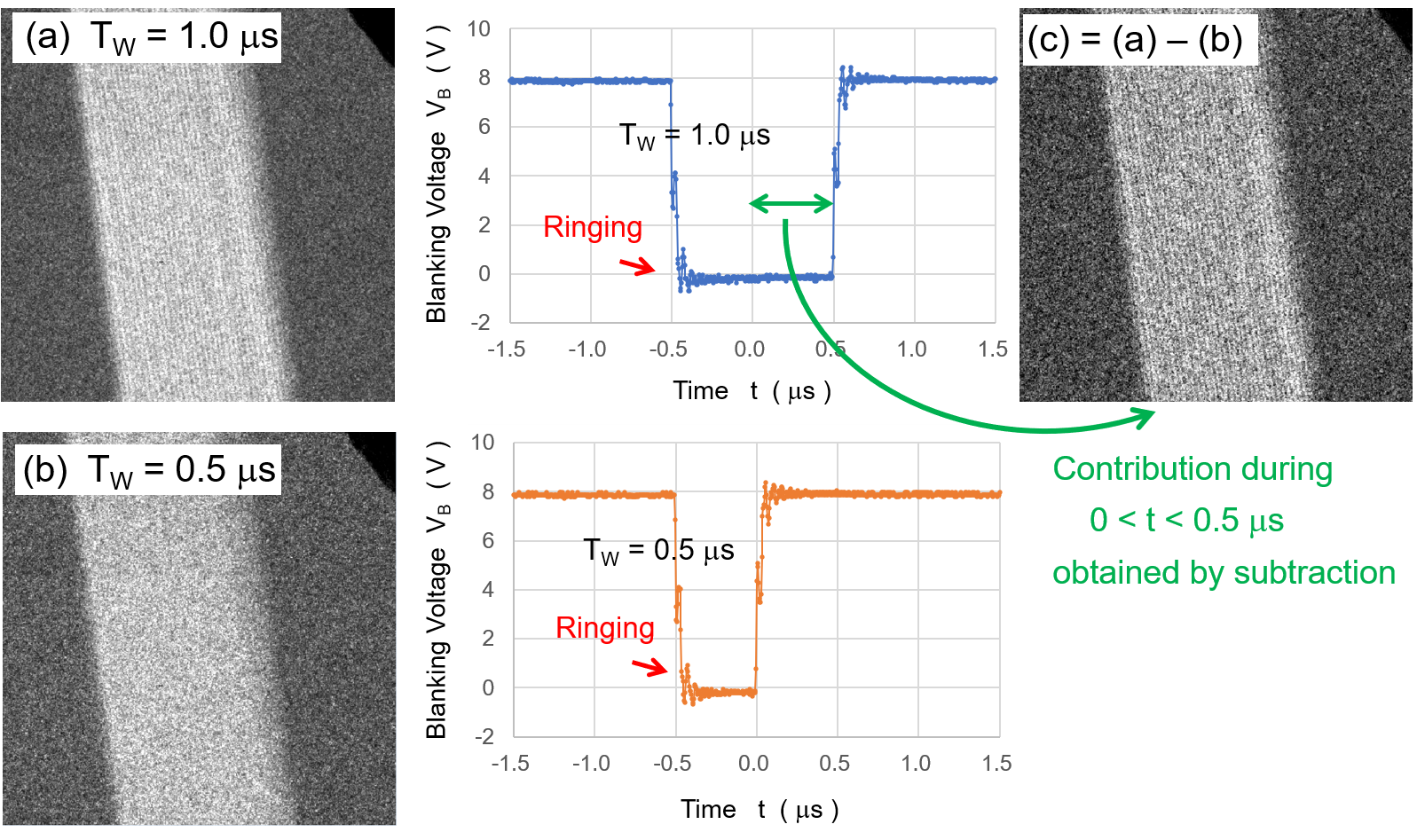

Supplement: dfad003_Supp [file dfad003_supp.zip › suppl_data/Fig. S2.tif]

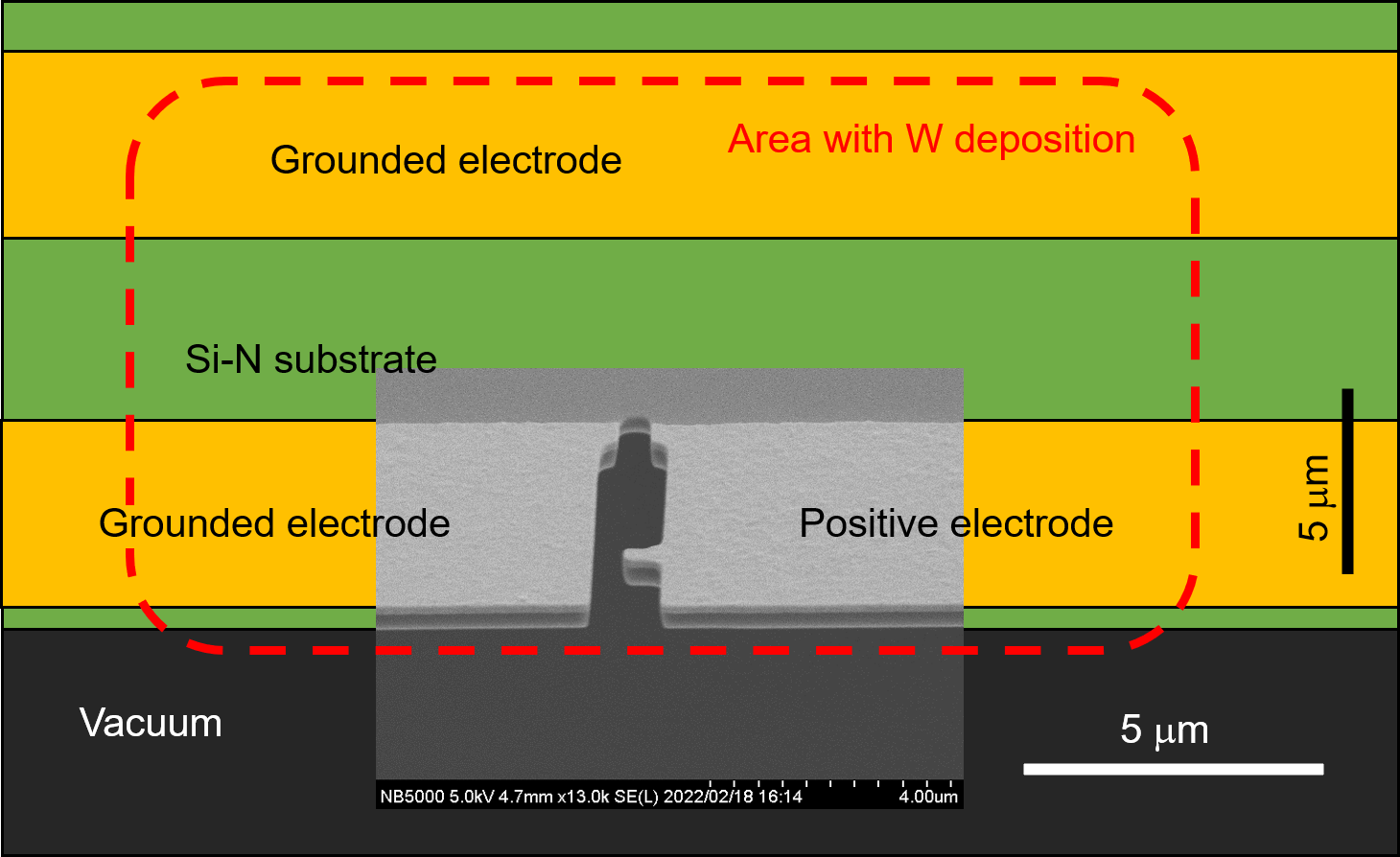

Supplement: dfad003_Supp [file dfad003_supp.zip › suppl_data/Fig. S3-1.tif]

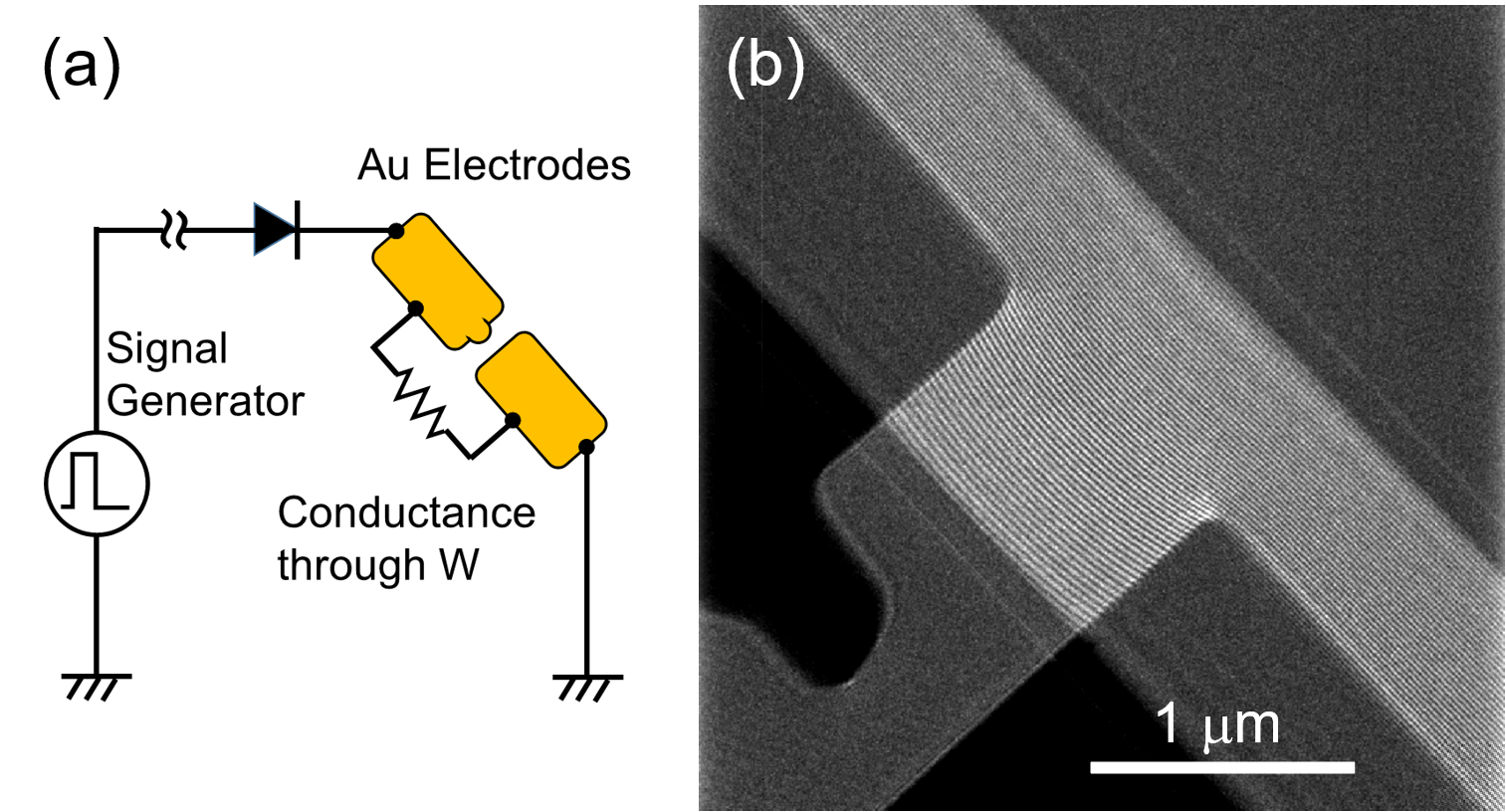

Supplement: dfad003_Supp [file dfad003_supp.zip › suppl_data/Fig. S3-2.tif]

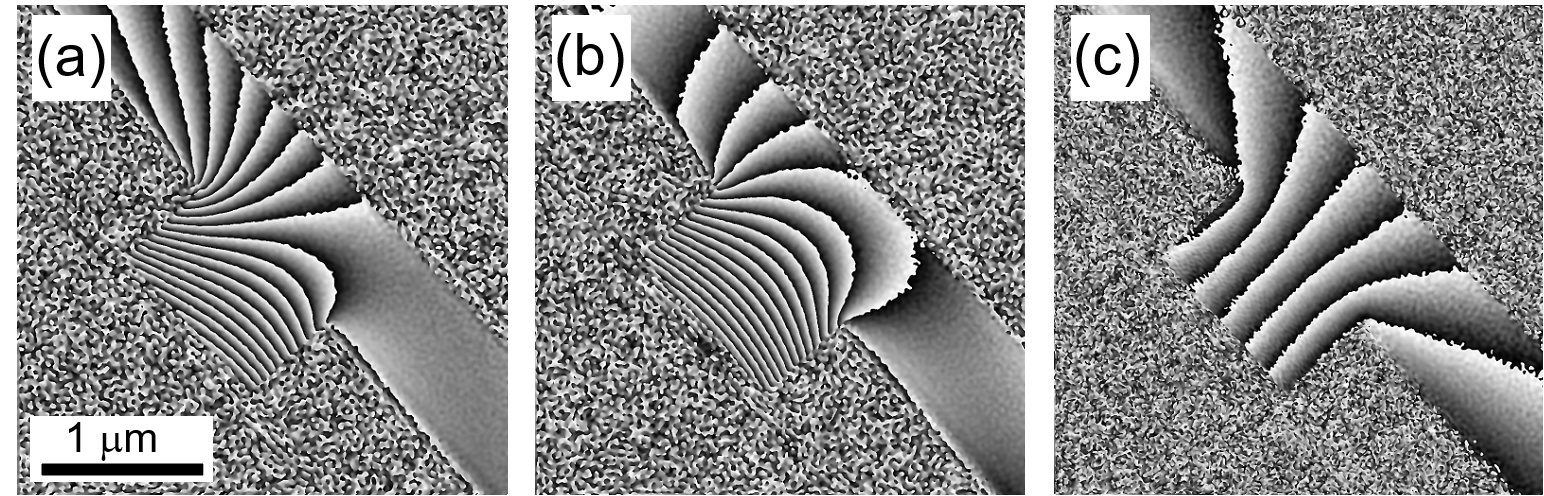

Supplement: dfad003_Supp [file dfad003_supp.zip › suppl_data/Fig. S4.tif]
